# Targeting WEE1 Inhibits Growth of Breast Cancer Cells That Are Resistant to Endocrine Therapy and CDK4/6 Inhibitors

**DOI:** 10.3389/fonc.2021.681530

**Published:** 2021-07-01

**Authors:** Yassi Fallah, Diane M. Demas, Lu Jin, Wei He, Ayesha N. Shajahan-Haq

**Affiliations:** ^1^ Department of Oncology, Lombardi Comprehensive Cancer Center, Georgetown University Medical Center, Washington, DC, United States; ^2^ Program in Genetics, Bioinformatics, and Computational Biology, VT Biological Transport, Virginia Tech, Blacksburg, VA, United States

**Keywords:** estrogen receptor positive breast cancer, endocrine therapy, drug resistance, CDK4/6 inhibitors, ribociclib, WEE1, AZD1775

## Abstract

Despite the success of antiestrogens in extending overall survival of patients with estrogen receptor positive (ER+) breast tumors, resistance to these therapies is prevalent. ER+ tumors that progress on antiestrogens are treated with antiestrogens and CDK4/6 inhibitors. However, 20% of these tumors never respond to CDK4/6 inhibitors due to intrinsic resistance. Here, we used endocrine sensitive ER+ MCF7 and T47D breast cancer cells to generate long-term estrogen deprived (LTED) endocrine resistant cells that are intrinsically resistant to CDK4/6 inhibitors. Since treatment with antiestrogens arrests cells in the G1 phase of the cell cycle, we hypothesized that a defective G1 checkpoint allows resistant cells to escape this arrest but increases their dependency on G2 checkpoint for DNA repair and growth, and hence, targeting the G2 checkpoint will induce cell death. Indeed, inhibition of WEE1, a crucial G2 checkpoint regulator, with AZD1775 (Adavosertib), significantly decreased cell proliferation and increased G2/M arrest, apoptosis and gamma-H2AX levels (a marker for DNA double stranded breaks) in resistant cells compared with sensitive cells. Thus, targeting WEE1 is a promising anti-cancer therapeutic strategy in standard therapy resistant ER+ breast cancer.

## Introduction

The majority of breast tumors are estrogen receptor alpha positive (ER+) and are clinically treated with endocrine therapy to deprive tumor cells of estrogen using aromatase inhibitors (AI) or to target the ER using tamoxifen or fulvestrant. In the adjuvant setting, tamoxifen can effectively reduce recurrences by 50% (1), however, 30-40% of patients will relapse within 15 years (2, 3). Hence, endocrine resistance remains a major clinical problem for ER+ breast cancer treatment. Estrogen drives cell cycle progression through transcriptional regulation of cyclinD1 (4). Therefore, inhibition of the cyclinD1/cyclin-dependent kinase 4 (CDK4) and CDK6 has been proposed as a rational therapeutic strategy for advanced or metastatic ER+ tumors (5). The development of highly selective, orally available and ATP competitive CDK4/6 inhibitors, palbociclib, ribociclib and abemaciclib has transformed the standard of care of ER+ and human epidermal growth factor receptor 2 negative (HER2-) metastatic breast cancer based on prolonged progression-free survival (PFS) when they are combined with antiestrogen therapies (6–8). Unfortunately, resistance to CDK4/6 inhibitors is inevitable. Approximately 20% of patients who progress on endocrine therapies show *de novo* or intrinsic resistance to CDK4/6 inhibitors and tumors that initially respond eventually acquire resistance to the combined therapies (9, 10). Loss or mutation of *RB1*, *PIK3CA* mutation, loss of CDK inhibitors such as *p16* or *p21*, loss of *FAT1* tumor suppressor, amplification of *CCNE1*, *FGFR1* or *CDK6* have been investigated as possible contributors to resistance to CDK4/6 inhibitors (11–19). However, our knowledge of how breast cancer cells develop resistance to CDK4/6 inhibitors is incomplete.

Antiestrogens and CDK4/6 inhibitors induce cycle arrest by suppressing multiple cyclins, such as cyclin D1, cyclin E1 or cyclin A2, that promote the G1/S transition (20, 21). Therapy induced defects in G1 checkpoint, e.g., due to faulty p53, a critical gatekeeper of the G1 phase, can drive cancer cells towards increased dependency on the G2 checkpoint to repair DNA damage (22), and thus, targeting G2 checkpoint has been proposed as an anti-cancer strategy in these cancer models (23). One such G2/M regulatory proteins is WEE1, a member of the tyrosine kinase family, that controls the timing of mitosis. WEE1 inhibits CDK1 by phosphorylating Tyr15 (Y15) and stops cells from entering mitosis to allow time for DNA repair (24, 25). In normal cells, the main function of WEE1 is to prevent replication of cells with DNA damage, however, in cancer cells, WEE1 has been linked to sustaining a tolerable level of genomic instability that favors tumor growth (24). Although poorly understood, subcellular localization of WEE1 protein may also play key regulatory roles at different stages of the cell cycle (26). Particularly in breast cancer, Murrow et al. used a RNAi screen of the human tyrosine kinome, to identify WEE1 as a potential therapeutic target in triple-negative breast cancer cells that lack ER, progesterone receptor [PR] or HER2 (27). In recent years, a number of preclinical studies have focused on understanding the functionality of WEE1 in breast cancer cells, particularly those with defective cell cycle regulation (28–30).

In this study, we used ER+ endocrine sensitive breast cancer cells, MCF7 and T47D, to generate long-term estrogen deprived (LTED) endocrine resistant breast cancer cells that are intrinsically resistant to CDK4/6 inhibitors. A small molecule inhibitor of WEE1, AZD1775 (Adavosertib) (31, 32), significantly decreased cell growth, increased G2/M cell cycle arrest and apoptosis in resistant cells compared with respective parental sensitive cells. Inhibition of p53 in endocrine sensitive cells showed increased sensitivity to AZD1775 suggesting that a defective p53 pathway may contribute to increased sensitivity to WEE1 inhibition in resistant cells. Furthermore, we showed that increased WEE1 gene expression in ER+ human tumors correlated with poor prognosis. Together, findings from our study supports the potential clinical use of anti-WEE1 therapy for ER+ breast tumors that have acquired resistance to endocrine therapy and are intrinsically resistant to CDK4/6 inhibitors.

## Materials and Methods

### Reagents

Ribociclib (LEE011), palbociclib (PD0332991), fulvestrant (ICI 182,780), tamoxifen (4-hydroxytamoxifen) and Adavosertib (AZD1775) were purchased from Selleck Chemicals (Houston, TX, USA). Abemaciclib (LY2835219**)** was purchased from Cayman Chemical Company (Ann Arbor, MI, USA). All other reagents were purchased from Sigma Aldrich (St. Louis, MO, USA). 4-Hydroxytamoxifen was dissolved in ethanol and all other drugs were reconstituted in dimethyl sulfoxide (DMSO). For *in vitro* assays, negative control (0.02%) was ethanol or DMSO.

### Cell Culture and Resistant Cell Line Establishment

ER+ breast cancer cell lines, MCF7 and T47D were obtained from Georgetown University Medical Center Tissue Culture Shared Resources and were maintained in humidified atmosphere with 5% CO_2_ at 37°C. MCF7 cell line was originally obtained from the Barbara A. Karmanos Cancer Institute, Detroit, MI, USA. Parental MCF7 and T47D were cultured in phenol red free IMEM (Improved Minimum Essential Medium), supplemented with 10% charcoal-stripped calf serum (CCS; Life Technologies, Grand Island, NY) and 10nM 17-beta-estradiol (E2). Long-term estrogen deprived (LTED) MCF7 and T47D variants were generated in cell culture by growing the cells in complete media without E2 for 10-12 months. LTED cells were maintained in E2-free complete media and all experiments were carried out in complete media. All cells were authenticated by DNA fingerprinting and tested regularly for Mycoplasma infection.

### Cell Proliferation Assay

Cells were seeded at various densities (5,000-1,2000 cells per well) in 96-well plastic tissue culture plates per cell line in addition to one extra 96-well plate for time=0 (t=0). T=0 time-point was added to some experiments because the basal rate of cell proliferation is reduced in LTED cells by about 30% compared with parental cells (**Supplementary Figures S1A, B**). The t=0 plate was stained with crystal violet (untreated) 24 h after plating and the remaining plates were dosed with 0.02% vehicle (DMSO or ethanol) or indicated drugs. Plates were ended at 72 h or 6 days. For all experiments longer than 72 h, media with respective treatments were replenished every 72 h. For crystal violet staining, plates were rinsed with 1xPBS to remove cellular debris. After, 100ul crystal violet was added to each well and incubated at room temperature for 1h. The stain was then removed and each plate was rinsed 4-8x with H_2_O to remove remaining stains. The plates were left to air-dry overnight, then were rehydrated with 100ul 0.1 M sodium citrate buffer in 50% ethanol and the plates were then measured using a V_Max_ kinetic microplate reader (Molecular Devices Corp., Menlo Park, CA) with an absorbance of 560 nm. For 12-day growth curve experiments, cells were seeded at 3-5 x 10^4^/well in 60mm^2^ dishes. At 24 h post plating, cells from one dish was used to measure cell number at t=0. For this, cells were trypsinized, suspended in PBS and cell number was measured using a Beckman Coulter Counter (Beckman Coulter Corp., Fullerton, CA, USA). Also, at 24 h post plating, all other dishes were treated with vehicle (DMSO or ethanol) or indicated treatments. Cells were counted every 3-days for 2 weeks. Each experiment had 3-6 technical replicates and all experiments were repeated three times.

### Immunoblotting and Antibodies

Cells were washed once with cold PBS and upon removal, cells were lysed in radio-immunoprecipitation assay (RIPA) buffer supplemented with PhosSTOP phosphatase and CompleteMini protease inhibitors (Roche, Switzerland) for protein extraction. Proteins were separated by polyacrylamide gel electrophoresis PAGE using 4-12% gradient gels followed by protein transfer onto nitrocellulose membranes with iBLOT2 (ThermoFisher Scientific, Waltham, MA). Membranes were then blocked in 5% nonfat dry milk in Tris- buffered saline with Tween-20 (TBST) and incubated at 4oC with primary antibodies. Proteins of interest were detected with horseradish peroxidase-conjugated secondary antibodies and Advansta WesternBright™ ECL Spray was used for detection (Thomas Scientific, Swedesboro, NJ). The following antibodies were purchased from Cell Signaling Technology (Danvers, MA): phospho-RB (Ser780) (#3590), RB (#9309), Cyclin D1 (#2978), phospho-CDK1 (Tyr15) (#4539), CDK1 (#77055), Wee1 (#13084), gamma-H2AX (Ser139) (#80312), H2aX (#7631) and cleaved PARP (Asp214) (#5625). Antibody to p53 (#ab32389) was purchased from Abcam (Cambridge, UK). For loading control, antibody to β-actin (#sc-47778) was purchased from Santa Cruz Biotechnology (Santa Cruz, CA).

### Transfection With WEE1 and p53 siRNA

Cells were plated at about 70% confluence in 6-well (for protein assessment) or 96-well (for cell proliferation assay) plates. The siRNAs (10nM, a mixture of 4 siRNA) were purchased from Dharmacon Inc. (Lafayette, CO) or scramble negative control were transfected into the cells using RNAiMAX (ThermoFisher Scientific, Waltham, MA). 72h post transfection (untreated), or 24h post transfection, vehicle (0.02% DMSO or ethanol) or different treatments as indicated, were added to the transfected cells and then cells were lysed and were subjected to western blot analysis. siRNA targeting the following sequences of Wee1: AAUAGAACAUCUCGACUUA; AAUAUGAAGUCCCGGUAUA; GAUCAUAUGCUUAUACAGA; CGACAGACUCCUCAAGUGA, and TP53: GAAAUUUGCGUGUGGAGUA; GUGCAGCUGUGGGUUGAUU; GCAGUCAGAUCCUAGCGUC; AGAAUAUUUCACCCUUC

### Cell Cycle and Apoptosis Assays

For cell cycle analysis, cells were plated at 1 x 10^6^/well in 10cm^2^ dishes. Cells were grown at 70% confluence in complete growth medium for 24 h. For measuring cell cycle profile under basal conditions cells were collected at 24, 48 or 72 h. For measuring cell cycle profile in response to drug treatment, cells were treated with vehicle, 500nM ribociclib, 500nM AZD1775 or subjected to E2 deprivation (cells were washed 3x with PBS and followed by adding media without E2) or the combination of these conditions as indicated for 6-days (with media change at 72 h). Cells were then fixed in ethanol, and analyzed by the Flow Cytometry Shared Resource according to the method of Vindelov et al. (33). For apoptosis assay, 2-5 x 10^5^/well cells plated in 6-well plates and were treated for 72 h, and stained with an Annexin V-fluorescein isothiocyanate and propidium iodide, respectively (Thermofisher Scientific Waltham, MA) according to the manufacturer’s protocol and fluorescence was measures by the Flow Cytometry Shared Resource at Georgetown University Medical Center. Each experiment was repeated at least three times.

### Estimates of Relapse-Free Survival and WEE1 Gene Expression Levels From Public Gene Expression Datasets

Publicly available datasets for gene expression from human ER+ breast cancer tumors were obtained: GSE2034 (34) and GSE7390 (35). Kaplan-Meier plots were generated using these datasets to estimate relapse-free survival over time (rfs_t) with indicated levels of WEE1 expression in their breast tumors. Graphs were generated using tools in the R statistical programming language.

### Statistical Analyses

Statistical analyses were performed using the Prism 8 (La Jolla, CA, USA). All experimental values were expressed as mean ± standard errors. Differences between two groups were determined by using the unpaired Student’s t-test, or ANOVA with a *post hoc* t-test for multiple comparisons, and p-values less than 0.05 were considered as statistically significant. The nature of interaction between E2-deprivation/fulvestrant, ribociclib or the combination with AZD1775 was calculated in MCF7, MCF7-LTED, T47D and T47D-LTED cells by the Highest Single Agent model (HSA) and Bliss score. The SynergyFinder R package was used to determine HSA and Bliss scores. A score >10 indicates a synergistic interaction, 10 to -10 indicates additivity and <-10 indicates antagonistic interaction (36).

## Results

### MCF7-LTED and T47D-LTED Cells Are Resistant to Antiestrogens and CDK4/6 Inhibitors

**Figure 1:**
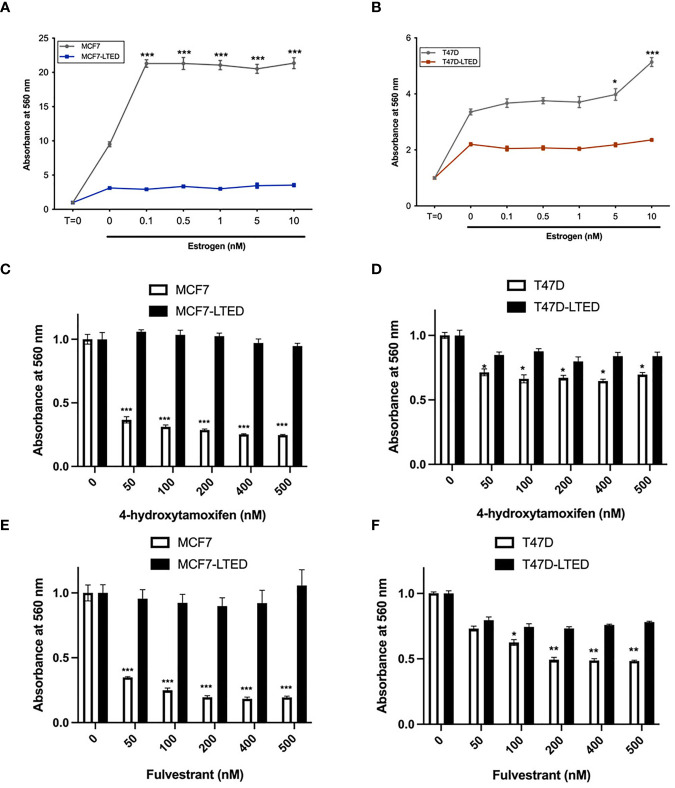
LTED cells are resistant to antiestrogens. **(A–F)** Parental cells (MCF7 and T47D) and their derivatives (MCF7-LTED and T47D-LTED) were treated with vehicle or increasing concentration of estrogen (17-estradiol; E2), tamoxifen (4-hydroxytamoxifen) or fulvestrant for 6 days (media was replenished at 72 h). Cell number was measured by crystal violet assays in 96-wells; absorbance was read at 560 nm. Points represent the mean ± SE of relative number (normalized to t=0 for estrogen and 0 nM for 4-hydroxytamoxifen and fulvestrant) for a single representative experiment performed in sextuplicate. *p < 0.05, **p < 0.01 and ***p < 0.001 (ANOVA) for cell number cell number at indicated drug concentrations compared with 0 nM for indicated cell lines. Data presented here is representation of three independent experiments (n=3).

**Figure 2:**
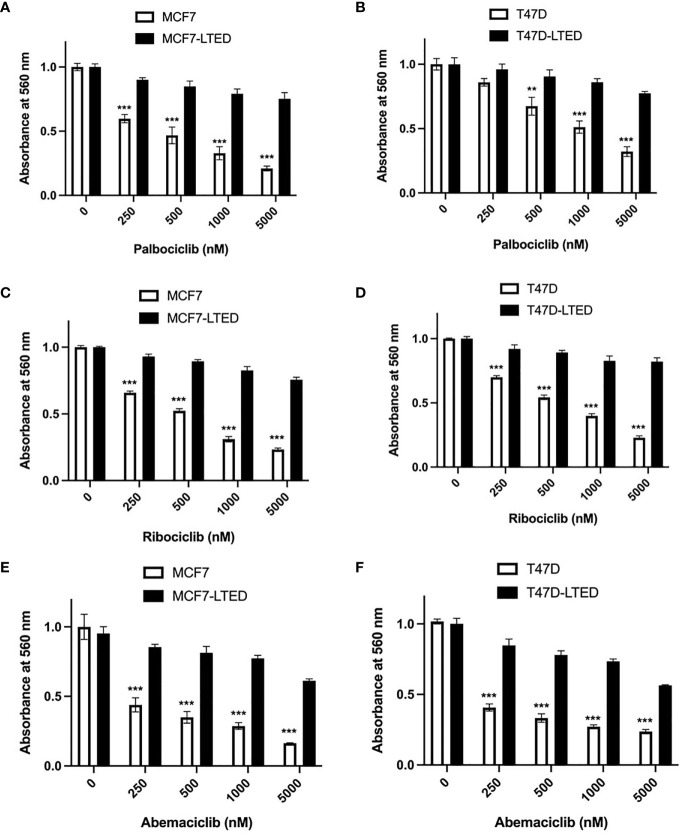
LTED cells are resistant to CDK4/6 inhibitors. **(A–F)** The growth rates of parental cells (MCF7 and T47D) and their derivatives (MCF7-LTED and T47D-LTED) were measured in presence of vehicle, palbociclib, ribociclib or abemaciclib for 6 days (media was replenished at 72 h). Cell number was measured by crystal violet assays in 96-wells; absorbance was read at 560 nm. Points represent the mean ± SE of relative number (normalized to 0 nM) for a single representative experiment performed in sextuplicate. **p < 0.01 and ***p < 0.001 (ANOVA) for cell number at indicated drug concentrations compared with 0 nM for indicated cell lines. Data presented here is representation of three independent experiments (n=3).

To determine antiestrogen sensitivity in parental ER+ MCF7 and T47D breast cancer cells and their respective LTED variants, MCF7-LTED and T47D-LTED cells, we measured cell proliferation over 6-days (media with respective treatment was replenished 72 h) with vehicle alone or increasing concentrations of 17beta-estradiol (E2), fulvestrant or tamoxifen (4-hydroxytamoxifen) (**Figures 1A–F**). Cell number increased significantly at 0.1 to 10 nM E2 in MCF7 cells and at 5 to 10 nM in T47D parental cells compared to 0 nM E2 (control). Cell number did not change with increasing levels of E2 in MCF7-LTED or T47D-LTED cells. Moreover, both MCF7-LTED (p<0.001) and T47D-LTED (p<0.5) cells showed significant decrease in sensitivity to 4-hydroxytamoxifen or fulvestrant compared with respective parental cells. Next, to compare the sensitivity of LTED and parental cells to CDK4/6 inhibitors, we treated the cells with either vehicle or increasing concentrations of each of the three CDK4/6 inhibitors: palbociclib, ribociclib or abemaciclib (**Figures 2A–F**). In both LTED variants, sensitivity to the CDK4/6 inhibitors was significantly (p<0.001) decreased compared to parental cells at respective concentrations of the drugs, which suggest that LTED cells are intrinsically resistant to CDK4/6 inhibitors.

**Figure 3:**
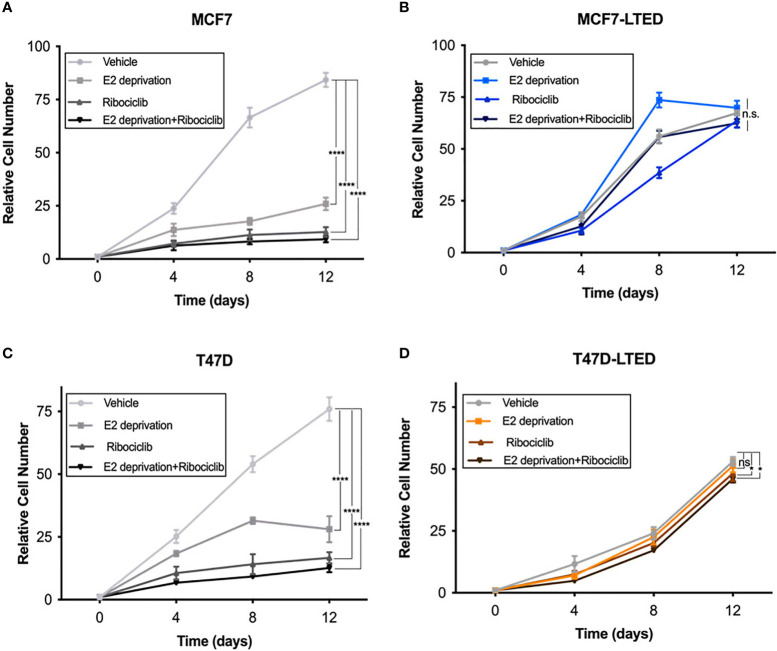
LTED cells are resistant to the combination of antiestrogens and CDK4/6 inhibitors. **(A–D) ** The growth rates of parental cells (MCF7 and T47D) and their derivatives (MCF7-LTED and T47D-LTED) treated with vehicle, E2 deprivation, 500nM ribociclib or the combination of E2 deprivation and ribociclib and cell number were measured for 12 days and compared to time=0 (start of treatment). Cells were suspended in PBS and counted by a Beckman Coulter Counter. Points represent the mean ± SE of relative number (normalized to t=0) for a single representative experiment performed in triplicate. *p < 0.05, ****p < 0.0001 and ns is not significant (ANOVA) for relative cell number with indicated treatment conditions at 12 days for respective cell lines. Data presented here is representation of three independent experiments (n=3).

Since antiestrogens are combined with CDK4/6 inhibitors in the clinic to treat advanced ER+ breast cancer, we sought to determine the effect of these drugs on cell proliferation in drug sensitive and resistant cells. For this, we measured cell proliferation in response to vehicle, E2 deprivation (to simulate aromatase inhibitor effect), ribociclib or the combination of E2 deprivation and ribociclib at 0, 4, 8 and 12 days (**Figures 3A, C**). Since LTED variants were resistant to all three CDK4/6 inhibitors (**Figure 2**), we selected ribociclib as the CDK4/6 inhibitor for the rest of the study. Cell proliferation was significantly inhibited in MCF7 and T47D parental endocrine sensitive cells when exposed to E2-deprivation (p<0.0001 and p<0.0001, respectively), 500nM ribociclib (p<0.0001 and p<0.0001, respectively) or the combination of E2-deprivation and ribociclib (p<0.0001 and p<0.0001, respectively) compared with their respective LTED variants (**Figures 3A, C**). These proliferation studies were repeated with fulvestrant in place of E2-deprivation (**Supplementary Figures S2A–D**) with similar results. In contrast, MCF7-LTED and T47D-LTED cells were resistant to fulvestrant, ribociclib or the combination compared with parental MCF7 or T47D cells, respectively, at day 12 (**Figures 3B, D**). Collectively, these data suggest that LTED derivatives have acquired resistance to antiestrogens but are also intrinsically resistant to CDK4/6 inhibitors.

**Figure 4:**
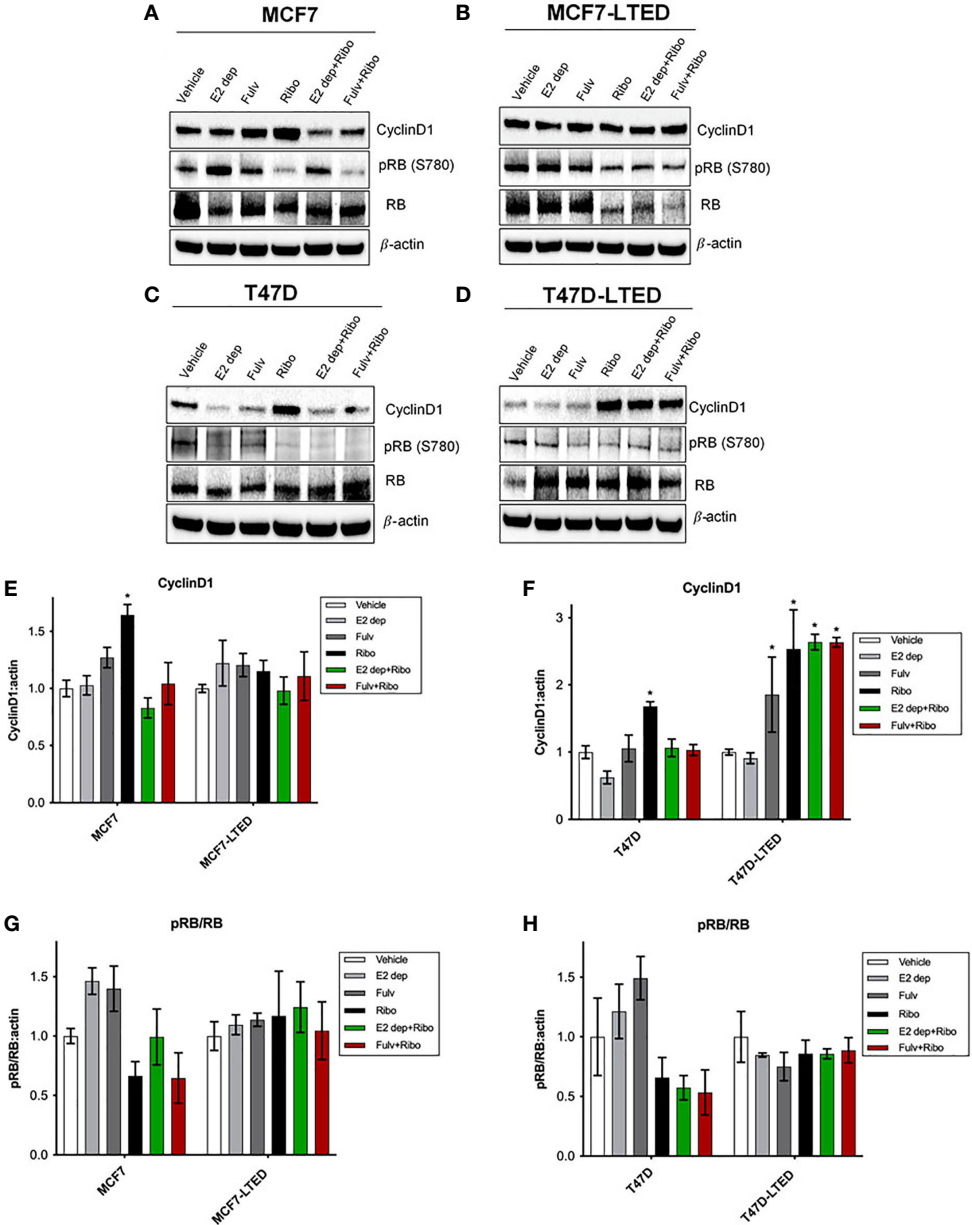
Differential levels of CyclinD1 and pRB(780) proteins in paternal and LTED breast cancer cells treated with combination of E2 deprivation and ribociclib. **(A, C)** Parental MCF7 and T47D and their derivative, **(B, D)** MCF7-LTED and T47D-LTED were treated with vehicle, E2 deprivation (E2 dep), 100nM fulvestrant (Fulv), 500nM ribociclib (Ribo) or the combination of E2 dep and ribo or fulvestrant and ribo. Total cell lysates were collected at 12 days and subjected to immunoblotting with indicated antibodies. Bar graphs show relative mean values of proteins levels + SE for cyclin D1 **(E, F)** and ratio of pRB(780) to total RB **(G, H)** following treatments compared with vehicle control for respective cell lines. Protein bands from immunoblots were measured and normalized to actin (loading control) from three independent (n=3) experiments using ImageJ software. *p < 0.05 (ANOVA) for respective protein levels for indicated conditions compared with corresponding vehicle controls.

Next, we assessed the protein levels cyclin D1 and phosphor-RB/total RB since these are key proteins involved in signaling associated with E2 or CDK4/6 responsiveness (37, 38). Western blot analysis on whole cell lysates isolated from cells treated with vehicle, ribociclib, E2-deprivation, fulvestrant or the combination or ribociclib with E2-deparivaiton or fulvestrant, at 12 days. In MCF7 and T47D cells, ribociclib treatment significantly (p=0.011 and p=0.007, respectively) increased cyclinD1 protein levels compared with vehicle (**Figures 4A, C, E, F**). In MCF7-LTED cells, cyclin D1 levels remained unchanged with any treatments compared to basal levels with vehicle alone. However, in T47D-LTED cells, ribociclib alone or in combination with E2-deprivation or fulvestrant (**Figures 4B, D–F**) significantly (p<0.05) increased cyclin D1 levels compared with vehicle though this increase in cyclin D1 did not correspond to changes in cell proliferation for T47D-LTED cells under these treatment conditions (**Figure 3D**). CDK4/6 complexes with cyclin D1 to phosphorylate and inactivate RB (39). Loss of RB expression is a commonly used criterion to exclude or include patients with advanced ER+ breast cancer on clinical trials with CDK4/6 inhibitors (21). Phospho-RB (S780) levels were decreased with ribociclib treatment in MCF7 and T47D parental cells but not in the LTED variants (**Figures 4A–D, G, H**). Together, these data suggest that cyclin D1 mediated signaling is differentially regulated in resistant LTED variants than in sensitive parental cells.

### G1 Phase of the Cell Cycle Is Prolonged in LTED Cells

**Figure 5:**
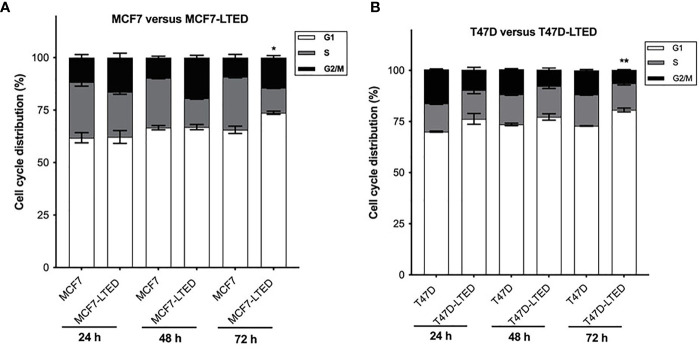
Increased G1 cell cycle arrest in LTED derivative cells. **(A, B)** Parental MCF7 and T47D and their LTED derivatives were harvested at 24, 48, and 72h and measured by fluorescence-activated cell sorting (FACS) cell cycle analysis. Bar graphs show mean percent cell cycle distribution with +SE *p < 0.05 and **p < 0.01 (Student’s t-test; n=3) for % cells in G1 at 72 h in respective LTED cells compared with parental cells.

Since LTED cells show a decreased rate of basal cell proliferation (**Supplementary Figure S1**), we determined whether cell cycle profiles are changed in LTED cells compared to parental cells. We compared cell cycle profiles in MCF7, MCF7-LTED, T47D and T47D-LTED cells at 24 h, 48 h, and 72 h. Interestingly, we observed a modest but significant increase in the G1 phase of the cell cycle at 72 h in both MCF7-LTED (p=0.0129) and T47D-LTED (p=0.0002) compared to parental cells (**Figures 5A, B**). These data suggest that both LTED cell variants may harbor a faulty G1 phases of the cell cycle that is dedicated to DNA replication and repair (40).

### AZD1775 Is Effective as a Monotherapy in LTED Cells

**Figure 6:**
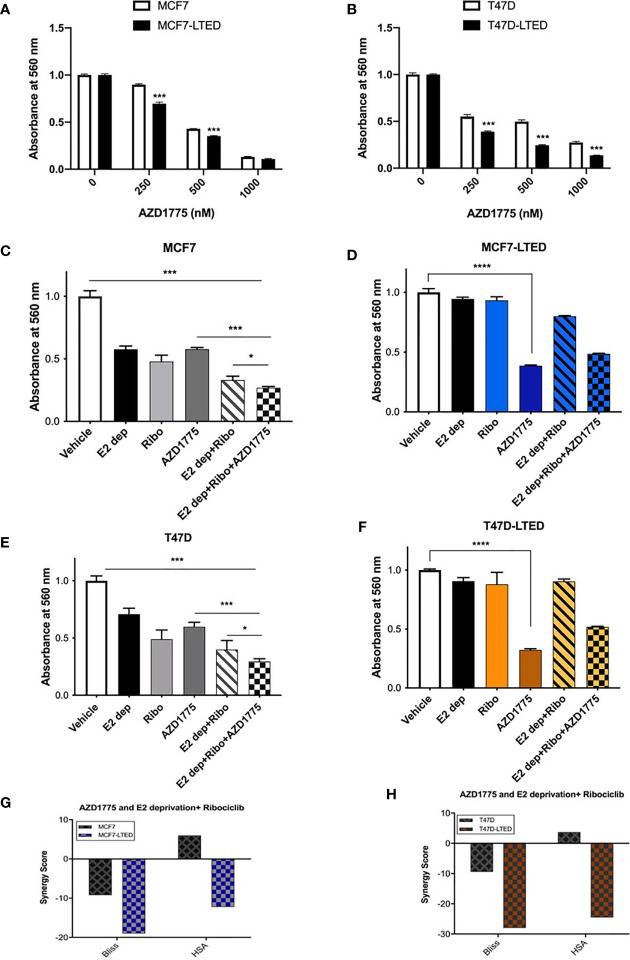
AZD1775 monotherapy suppressed growth of LTED cells. **(A, B)** Parental cells (MCF7 and T47D) and LTED derivatives (MCF7-LTED and T47D-LTED) were treated with indicated concentration of AZD1775. **(C–F)** MCF7 and T47D cells and their LTED cells were treated with E2 deprivation (E2 dep), 500nM ribociclib (Ribo), 500nM AZD1775, combination of E2 dep and ribo or the triple combination of E2 dep, ribo and AZD1775 for 6 days. Inhibition of proliferation was measured by crystal violet assays. Data represents the average value ± SE of six replicates of relative number (normalized to vehicle alone). *p < 0.05, ***p < 0.001 and ****p < 0.0001 (ANOVA) for cell number for indicated treatment conditions compared to conditions designated by lines. **(G, H)** The Bliss and HSA scores using the SynergyFinder R package was used. A score >10 indicates a synergistic interaction, 10 to -10 indicates additivity and <-10 indicates antagonistic interaction between combination of E2 dep+ribo and AZD1775. All experiments were repeated at least three times.

Cancer cells with impaired G1 checkpoint function show increased sensitivity to AZD1775, a clinical grade small molecule inhibitor of WEE1 kinase, that targets the G2 checkpoint (41). Therefore, we compared the efficacy of AZD1775 in inhibiting cell proliferation in endocrine sensitive and resistant cells. Cells treated with increasing concentrations (250nM to 1000nM) of AZD1775 at 72 h showed significantly more decrease in cell proliferation in LTED variants of both cell types compared with parental cells (p<0.0001) (**Figures 6A, B**). We further confirmed the increased dependency on WEE1 for cell proliferation in LTED cells compared with parental cells using WEE1 siRNA. Knockdown of WEE1 decreased cell proliferation by ≥50% in LTED cells and only by 25% in parental cells compared with controls condition (**Supplementary Figures S4A–D**). Cells transfected with WEE1 siRNA showed decreased WEE1 protein and increased inhibition of p-CDK1(Y15) (**Supplementary Figures S4E, F**). Next, we compared cell proliferation in all cells with AZD1775 alone or in combination with E2-deprivation, fulvestrant, ribociclib or the combination of E2-deprivation and ribociclib or fulvestrant and ribociclib at 6 days (**Figures 6C–F**). As expected, cell proliferation in both MCF7 and T47D cells was significantly decreased with E2 deprivation alone (p<0.0001, and p<0.0001, respectively) and 500nM ribociclib alone (p<0.0001 and p<0.0001, respectively, **Figures 6C, E**). Furthermore, in MCF7 and T47D cells, combination of E2 deprivation and 500nM ribociclib significantly decreased cell proliferation (p<0.0001 and p<0.0001, respectively) compared to vehicle treated cell (**Figures 6C, E**). Similar results were obtained when we repeated the same experiment in MCF7 and T47D parental cells with 100nM fulvestrant in place of E2-deprivation (**Supplementary Figures S3A–D**). However, in the LTED variants, there was no differences in cell proliferation with ribociclib, E2-deprivation or fulvestrant or the combination compared with vehicle treated cells (**Figures 6D, F**). AZD1775 as a single agent significantly inhibited cell proliferation in parental cells and LTED variants (p<0.0001, **Figures 6C–F**). The HSA and Bliss scores were between -10 and 10 for both MCF7 and T47D cells, which suggest an additive effect between AZD1775 and ribociclib plus E2-deprivation. For both MCF7-LTED and T47D-LTED variants, HSA and Bliss scores were >-10, which suggest an antagonist interaction of AZD1775 and ribociclib plus E2-deprivation combination (**Figures 6G, H**). Together, these cell proliferation studies suggest that AZD1775 is effective as a monotherapy for inhibiting cell growth in endocrine resistant and CDK4/6 inhibitor cross-resistant breast cancer cells.

### AZD1775 Induces G2/M Cell Cycle Arrest and Apoptosis in LTED Cells

**Figure 7:**
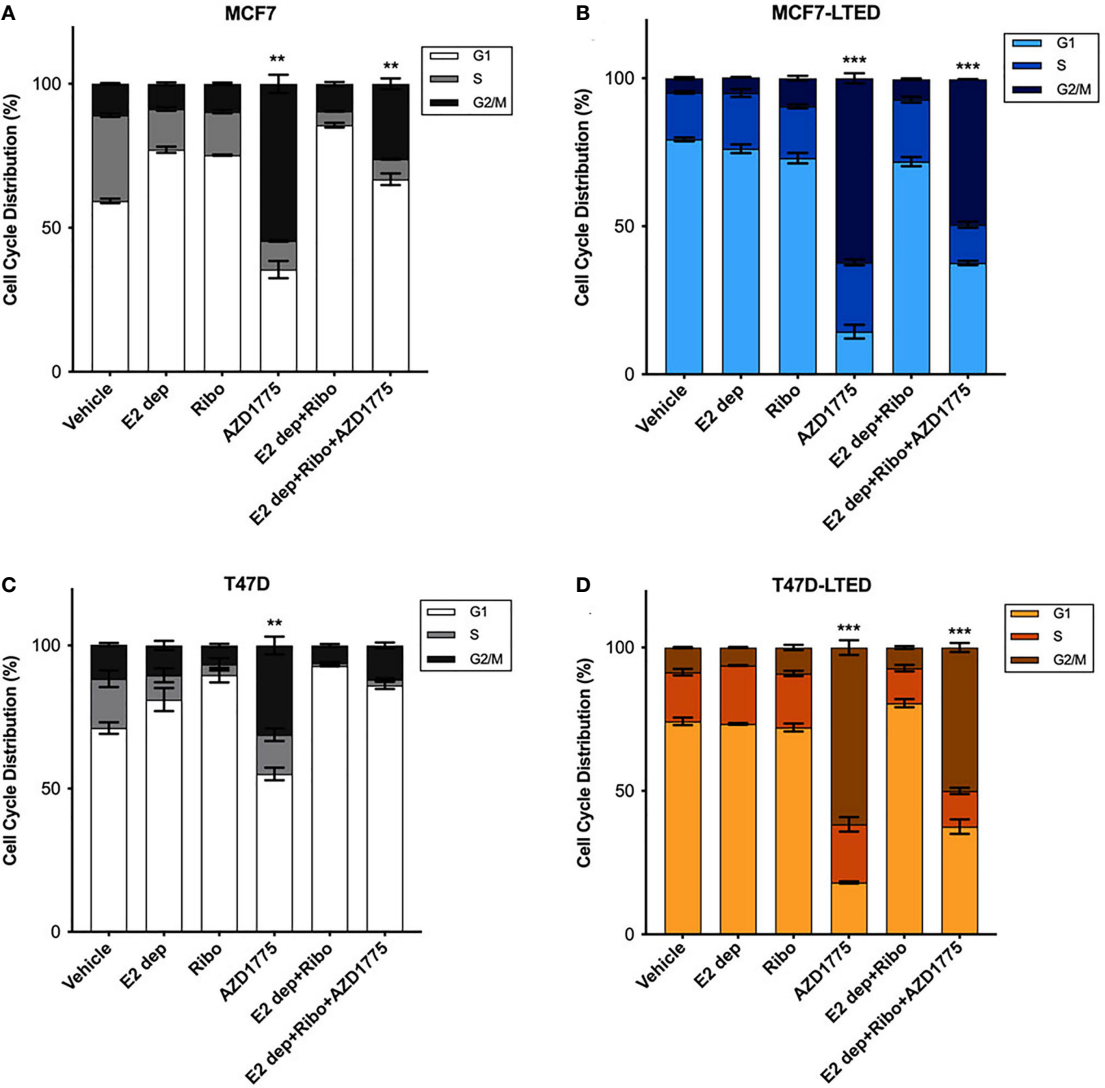
Inhibition of WEE1 with AZD1775 increased G2/M cell cycle arrest in LTED cells. **(A–D)** MCF7 and T47D and their LTED derivatives were treated with vehicle, E2 deprivation (E2 dep), 500nM ribociclib (ribo), 500nM AZD1775, combination of E2 dep and ribo or the combination of E2 dep, ribo and AZD1775 for 6 days. Cell cycle profiles under different treatment conditions were analyzed by fluorescence-activated cell sorting (FACS) and compared to vehicle treated cells. Results are reported as mean percent cell cycle distribution + SE **p < 0.01 and ***p < 0.001 (ANOVA) for % cell in G2/M phase for indicated treatment conditions compared with vehicle control in respective cell lines. Bar graphs represent data from three independent experiments (n=3).

AZD1775 treatment in cancer cells in known to induce G2/M cell cycle arrest (42, 43). To evaluate the effect of AZD1775 on cell cycle and correlate them with anti-proliferative effects of the drug, we treated both pairs of sensitive and resistant cells with vehicle alone, ribociclib, E2-deprivation, AZD1775 and the combination of ribociclib, E2-deprivation and AZD1775 for 6-days and measured cell cycle profile with flow cytometry (**Figure 7**). As expected, MCF7 (p<0.001 and<0.001, respectively) and T47D (p<0.01 and p<0.001, respectively) parental cells treated with ribociclib or subjected to the combination of E2 deprivation and ribociclib showed a significant increase in the proportion of cells arrested in the G1 phase of the cell cycle compared with vehicle treated cells (**Figures 7A, C**). Cell cycle profiles of LTED variants did not show an increase with either E2-deprivation or the combination of E2 deprivation and ribociclib when compared with vehicle (**Figures 7B, D**). In both parental and LTED cells, treatment with AZD1775 induced significant (p<0.0001) increase in G2/M cell cycle arrest compared with respective vehicle controls. In LTED cells, >60% of cells arrested in G2/M with AZD1775 treatment compared with ≤50% in parental cells. Taken together, we show that cell cycle profiles in LTED cells are unchanged with E2 deprivation or ribociclib but shift to G2/M arrest with AZD1775 treatment.

**Figure 8:**
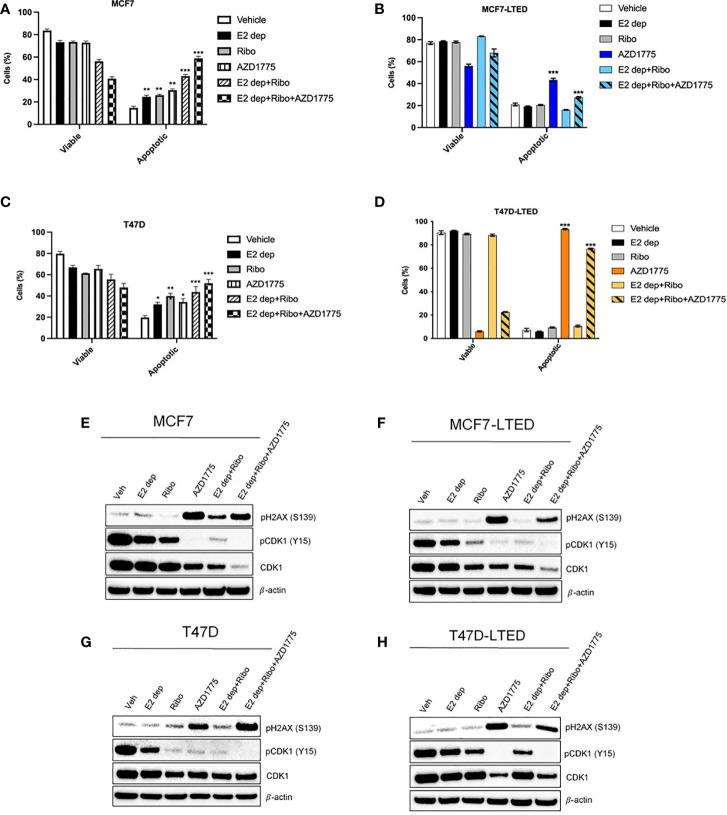
AZD1775 induced marked increase in apoptosis in LTED cells. **(A–D)** Parental MCF7 and T47D cells and their corresponding LTED derivatives were treated with vehicle, E2 deprivation (E2 dep), 500nM ribociclib (ribo), 500nM AZD1775 alone or the combination of E2 dep, ribo or the triple combination of E2 dep, ribo and AZD1775 for 6 days. Annexin V-FITC assay was used to measure apoptosis levels. *p < 0.05, **p < 0.01 and ***p < 0.001 (ANOVA) for % apoptotic cells with different treatment conditions compared with vehicle alone for respective cell lines. **(E–H)** Cells were treated with indicated treatments for 6 days. Whole cell lysates were subjected to immunoblotting with indicated antibodies; β-actin was used a loading control. All blots and proliferation assays are from three independent experiments (n=3).

To compare the effect of AZD1775 as a single agent or in combination with E2-deprivation and ribociclib on cell survival, we measured cell death *via* apoptosis by staining cells with Annexin V-fluorescein isothiocyanate followed by analysis by flow cytometry in treated cells at 6-days. We observed a significant increase apoptotic cells with E2-dprivation, ribociclib or the combination in both sensitive MCF7 (p<0.01, p<0.01, and p<0.0001, respectively) and T47D cells (p<0.05, p<0.01 and p<0.0001, respectively) compared with corresponding vehicle treated cells (**Figures 8A, C**). In contrast, we did not observe significant apoptosis levels with E2-deprivation, ribociclib or the combination in resistant MCF7-LTED and T47D-LTED cells (**Figures 8B, D**). However, there was a significant increase in apoptosis in MCF7-LTED or T47D-LTED with AZD1775 treatment (p<0.0001; p<0.0001, respectively, **Figures 8B, D**) compared to parental cells. Furthermore, treatment with E2-deprivation and ribociclib in combination with AZD1775 significantly increased apoptosis in MCF7-LTED and T47D-LTED (p<0.0001 and p<0.0001 respectively), however, the level of apoptosis in cells was decreased by about 10% when ribociclib, E2-deprivation and AZD1775 were combined compared to AZD1775 alone (**Figures 8B, D**). Induction of apoptosis with different treatments compared with vehicle was confirmed using cleaved PARP (poly-ADP ribose polymerase), a marker of apoptosis induction (**Supplementary Figure S5**). Together, these data suggest that in LTED cells, AZD1775 alone induced marked increase in cell death *via* apoptosis.

Immunoblotting was performed to evaluate the mechanism of AZD1775 alone or in combination with E2-dperivation and ribociclib in both sensitive or resistant cells. Following 6-days of exposure to the drugs, all cells demonstrated marked decrease in p-CDK1 (Y15) with AZD 1775 alone and this decrease was maintained in combination with E2 deprivation and ribociclib (**Figures 8E–H**). Clinical work has shown DNA double stranded break (DSB) is commonly detected with phosphorylation of histone H2AX (phospho-H2AX[S139]; gamma-H2AX) with AZD1775 treatment (44, 45). Consistent with previous findings, we found an increase in gamma-H2AX in cells treated with AZD1775 alone or in combination with ribociclib and E2-deprivation in both MCF7 and T47D cells and this increase was enhanced in MCF7-LTED and T47D-LTED cells (**Figures 8E–H**). However, level of gamma-H2AX was slightly decreased when cells were treated with E2-deprivation, ribociclib and AZD1775 compared with AZD1775 alone, which complemented our cell growth results and collectively showed that AZD1775 is more potent in inhibiting cell growth LTED cells as a monotherapy.

### Knockdown of p53 Enhances AZD1775 Mediated Inhibition of Cell Growth in Parental MCF7 and T47D Cells

**Figure 9:**
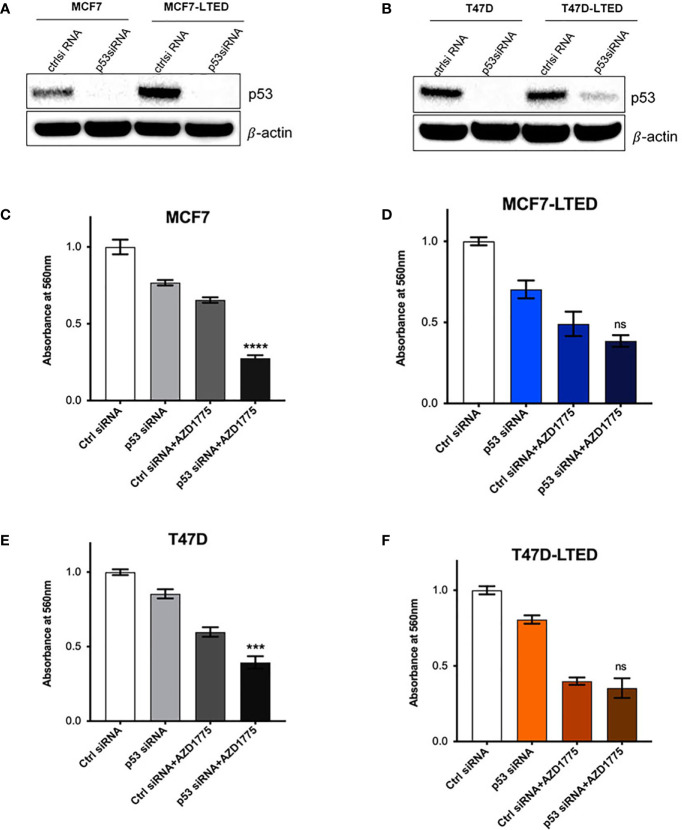
Knockdown of p53 enhances AZD1775 mediated inhibition of cell growth in parental MCF7 and T47D cells. **(A, B)** Parental MCF7 and T47D and their respective LTEDs were transfected with p53 siRNA or scramble negative control (ctrl) siRNA for 72h. Whole cell lysates were subjected to immunoblotting with p53 antibody to confirm knockdown; β-actin was used as protein loading control. **(C–F)** Following 24h post transfection, cells were treated with 500nM AZD1775 for another 48 h and cell number was measured using the crystal violet assay. ***p < 0.001, ****p < 0.0001 or ns (not significant) (ANOVA) for cell number with p53 siRNA+AZD1775 compared to Ctrl siRNA+AZD1775 for respective cell lines. All blots and proliferation assays are from three independent experiments (n=3).

Cancer cells that harbor TP53 mutations show increased sensitivity to AZD1775 (46, 47). T47D cells are known to contain a missense mutation (L194F) in TP53 that contributes to increased stability of p53 protein compared with MCF7 cells that contain wild-type *TP53* (48). To confirm a possible role for p53 activity in AZD1775 sensitivity in LTED variants, we tested whether inactivation of p53 gene in the various cell lines affects sensitivity to AZD1775 (**Figure 9**). We transfected MCF7, MCF7-LTED, T47D and T47D-LTED cells with siRNA to p53 or non-specific control (ctrl) sequences for 24 h followed by treatment with AZD1775 for 48 h. Western blot analysis of p53 protein levels confirmed successful knockdown of p53 gene in cells transfected with p53 siRNA compared with cells transfected with ctrl siRNA (**Figures 9A, B**). Measurement of cell proliferation showed significant increase in sensitivity to AZD1775 in both MCF7 and T47D cells transfected with p53 siRNA compared with their LTED counterparts (P<0.0001 and p<0.001 respectively, **Figures 9C, E**). However, knockdown of p53 in MCF7-LTED and T47D-LTED cells did not further increase sensitivity to AZD1775. Thus, these data suggested that sensitivity to WEE1 inhibition is dependent on p53 function in sensitive parental cells, however, this p53-dependence is lost in resistant LTED cells.

### Increased WEE1 Correlate With Survival in LTED Cells and With Poor Prognosis in ER+ Human Tumors

**Figure 10:**
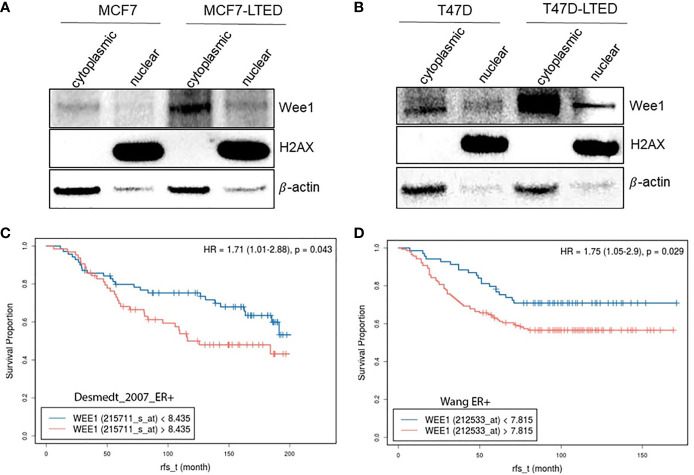
Increased WEE1 correlates with survival of LTED cells and with poor prognosis in breast cancer. **(A, B)** Protein samples from MCF7 and T47D and their LTED derivatives (MCF7-LTED and T47D-LTED) were collected under basal conditions and fractionated to nuclear or cytoplasmic fractions and immunoblotted with indicated antibodies. Increased WEE1 protein levels was detected in both cytoplasm or nuclei of MCF7-LTED cells compared with the corresponding fractions in MCF7 cells. Similarly, in T47D-LTED cells, WEE1 protein levels were increased in cytoplasmic and nuclear fractions compared with the corresponding fractions in T47D cells. Total H2AX (nuclear fractions) and β-actin (cytosolic fractions) were used as controls. Blot represents one of three independent experiments. **(C, D)** Kaplan-Meier survival curves was generated using two databases (GSE2034; n=134, GSE7390; n=209). Higher WEE1 gene expression (red) correlated with reduced relapse free survival (rfs) compared with low WEE1 gene expression (blue).

To investigate whether the LTED variants of these cells show altered WEE1 protein levels, we determined basal WEE1 protein levels in nuclear or cytoplasmic fractions from MCF7, MCF7-LTED, T47D and T47D-LTED cells that were 70-80% confluent (**Figure 10**). Nuclear-to-cytoplasmic cycling of important G2 checkpoint proteins such as WEE1 may be a key mechanism of G2 checkpoint regulation (49–51). Levels of WEE1 proteins were higher in both cytoplasmic and nuclear fractions of MCF7-LTED and T47D-LTED compared with parental cells. Thus, increased WEE1 protein levels correlates with resistance to antiestrogens or CDK4/6 inhibitors in LTED cells. To determine whether WEE1 gene expression was associated with survival, we analyzed publicly available gene expression datasets (**Figures 10C, D**) for ER+ breast tumors. Relapse-free survival over time (rfs_t) was estimated by Kaplan-Meier plots from two databases (GSE2034, GSE7390) showed that high WEE1 gene expression significantly correlated with unfavorable prognosis in lymph node negative ER+ breast tumors. Although this data suggests a role of increased WEE1 in ER+ breast cancer progression, further studies are needed to determine whether increased WEE1 expression correlate with resistance to antiestrogens or CDK4/6 inhibitors.

## Discussion

Breast tumors that progress on endocrine and CDK4/6 inhibitor therapies are often treated with combination of antiestrogens and mTOR inhibitors or with chemotherapies with nominal success (9, 52). Recently, models of ER+ breast cancer cells are being explored to elucidate resistant mechanisms in order to identify targeted therapies for endocrine and CDK4/6 inhibitors resistant breast tumors (13, 53). WEE1 is a tyrosine kinase that regulates G2/M checkpoints and timing of mitosis in normal cells (54). Inhibitory phosphorylation of CDK1 (on Y15), delays cells from entering mitosis and allows time for DNA repair. Therefore, cancer cells with defective G1 checkpoint, rely on G2 checkpoint for DNA repair and targeting WEE1 in these cells induce untimely mitosis and cell death (41). In this study, we used endocrine sensitive and resistant breast cancer cell line pairs to evaluate whether the WEE1 inhibitor, AZD1775, as a single agent or in combination with antiestrogens or CDK4/6 inhibitors, is a reasonable therapy option for inhibiting growth of resistant cells. We found that AZD1775 is effective as a single agent in inhibiting growth in resistant cells. Using siRNA, we show that cell proliferation in antiestrogen and CDK4/6 inhibitor resistant LTED cells are significantly more dependent on WEE1 compared sensitive parental cells. Combining AZD1775 with antiestrogen or E2-deprivation did not increase inhibition potential of this drug. Furthermore, we show that AZD1775 decreased cell survival in resistant cells by inducing apoptosis and G2/M cell cycle arrest.

Combination of AZD1775 with a DNA-damaging chemotherapy agent has shown increased efficacy of both drugs in various preclinical cancer models (28, 55–57). In breast cancer, targeting WEE1 with AZD1775 has been recently investigated by others as a promising strategy in combination therapeutic approaches in different subtypes of the disease, particularly in preclinical models of TNBC (28, 30, 58–60). In HER2-positive breast cancer cells models, treatment with AZD1775 overcomes resistance to standard-of-care therapy trastuzumab, a monoclonal antibody that targets HER2 (43). In ER+ breast cancer cell models that were specifically made resistant to ribociclib, combination of AZD1775 and ribociclib inhibited proliferation in resistant cells (61). In comparison, our MCF7-LTED and T47D-LTED breast cancer cell models represent acquired endocrine resistant breast cancer that are intrinsically resistant to CDK4/6 inhibitors, which comprises about 20% of breast cancer patients in the clinic (9). Treatment with combination of standard-of-care therapy, antiestrogen (E2-deprivation or fulvestrant) plus ribociclib and AZD1775 did not show increased inhibition of cell proliferation compared with treatment with antiestrogen or AZD1775 alone (**Figures 6C–H**). Currently, advanced ER+ tumors that progress on antiestrogens and CDK4/6 inhibitors are treated with antiestrogens combined with mTOR or PI3K kinase inhibitors (9). Based on our studies, WEE1 with AZD1775 is a potential therapeutic option for some therapy resistant ER+ tumors, although more investigation is needed to identify biomarkers for selection of tumors that will respond to WEE1 inhibitors. Increased gamma-H2AX has been proposed as a marker of sensitivity to WEE1 inhibitors (45). In our standard-of-care therapy resistant ER+ breast cancer cells, treatment with AZD1775 increased gamma-H2AX as a single agent but this signal was diminished when AZD1775 was combined with E2-deprivation/fulvestrant and ribociclib (**Figures 8H, F**). Therefore, AZD1775 is effective as a single agent in ER+ breast cancer cells that are resistant to antiestrogen and CDK4/6 inhibitors but the anti-proliferative effect of WEE1 inhibition in these cells is hampered when combined with antiestrogens or CDK4/6 inhibitors.

G1 checkpoint can be deregulated in cancer cells with p53 mutation and these cells show increased sensitivity to WEE1 inhibition by prematurely entering G2 and promoting apoptosis (25, 47). This mode of synthetic lethality in tumors with weakened p53 could be exploited with a WEE1 inhibitor such as AZD1775 (62). In this study, we show that knockdown of p53 in sensitive parental cells, but not in resistant cells, significantly increased sensitivity to AZD1775 (**Figures 9C, E**). MCF7 cells are wild-type for p53 while T47D cells have been reported to contain a mutation leading to an amino acid change (L194F) (63). However, sensitivity to AZD1775 is not notably different in MCF7 cells compared with T47D cells, and downregulation of p53 in both of these cell lines increased sensitivity to AZD1775. Therefore, although p53 mutations can increase sensitivity to WEE1 inhibition, L194F mutation in T47D cells may not be sufficient to increase sensitivity to AZD1775. On the other hand, downregulation of p53 in MCF7-LTED and T47D-LTED variants did not further accentuate sensitivity to AZD1775. Therefore, it is likely that in LTED cells, p53 may be altered, possibly due to post-translational modifications that suppressed normal p53 function, and consequently knockdown of p53 does not further affect sensitivity to AZD1775 in these cells. Additional studies to characterize the nature of p53 modifications in LTED cells are currently underway in our laboratory.

AZD1775 is currently being evaluated in clinical trials for the treatment of various cancers in combination with other anticancer therapies or as a single agent (44, 64). While improved survival and manageable side effects were observed with AZD1775 in these clinical trials, our understanding of useful biomarkers to improve efficacy is incomplete. The preclinical data presented in this study provides a rationale for using WEE1 inhibitors as a promising novel therapy for endocrine resistant and CDK4/6 inhibitor resistant breast cancer. Further studies including *in vivo* models are needed to validate the use of AZD1775 and identity biomarkers of its efficacy in this disease setting.

## Conclusions

The goal of this study was to investigate whether inhibition of WEE1 is an effective therapy for breast cancer cells that have progressed on endocrine therapies and are intrinsically resistant to CDK4/6 inhibitors. Based on the findings from this study, we provide a convincing rationale for using AZD1775 as a monotherapy in advanced ER+ breast cancer. However, further investigation is warranted to identity biomarkers that could better guide patient selection.

## Data Availability Statement

The datasets utilized in this study can be found in online GEO repositories - GSE2034: https://www.ncbi.nlm.nih.gov/geo/query/acc.cgi?acc=gse2034; GSE7390: https://www.ncbi.nlm.nih.gov/geo/query/acc.cgi?acc=gse7390.

## Author Contributions

Conceptualization, YF and ASH. Methodology, YF. Performing experiments and analysis, YF and DD. Formal analysis and software, LJ and WH. Writing—review and editing, YF and ASH. Project administration, ASH. All authors contributed to the article and approved the submitted version.

## Funding

This research was partly funded by Public Health Service grant R01-CA201092 to ASH. YF was supported by a Susan G Komen TREND grant (GTDR15330383). Technical services were provided by the following shared resources at Georgetown University Medical Center: Tissue Culture and Flow Cytometry Shared Resources that were funded through Public Health Service award 1P30-CA-51008 (Lombardi Comprehensive Cancer Center Support Grant).

## Conflict of Interest

The authors declare that the research was conducted in the absence of any commercial or financial relationships that could be construed as a potential conflict of interest.
