# Supplementary material for: Targeting WEE1 Inhibits Growth of Breast Cancer Cells That Are Resistant to Endocrine Therapy and CDK4/6 Inhibitors
Source: Front Oncol. 2021 Jul 1;11:681530. doi: 10.3389/fonc.2021.681530 (PMC8281892; doi:10.3389/fonc.2021.681530)
Supplement: Supplementary file 1 [file DataSheet_1.pdf]

Supplementary Figure S1

A

B

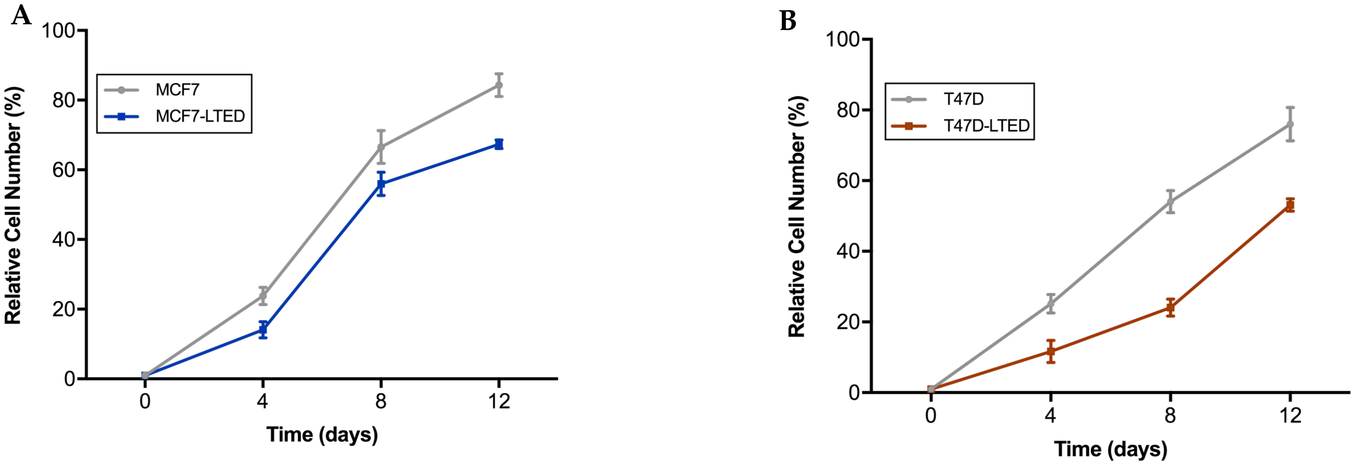

Figure S1. Basal growth rate of LTED cells. (A, B) The growth rates of estrogen receptor positive (ER+) parental cells (MCF7 and T47D) and their derivatives (MCF7-LTED and T47D-LTED) were trypsinized, suspended in PBS and cell number was measured using a Beckman Coulter Counter at 0, 4, 8 and 12 days. Points represent the mean ± SE of relative number (normalized to t=0) for a single representative experiment performed in triplicates. Data presented here is representation of three independent experiments (n=3).

Supplementary Figure S2

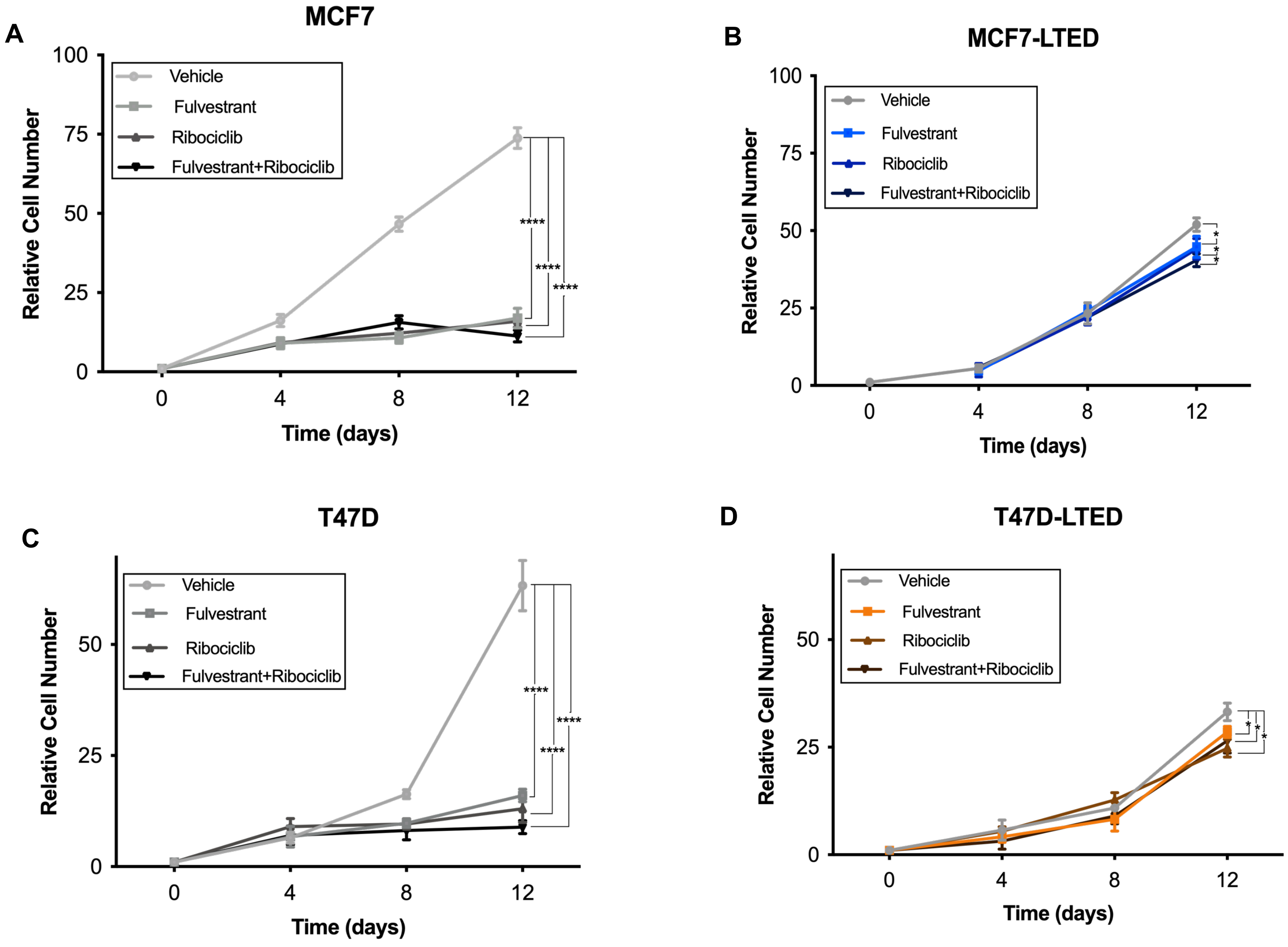

Figure S2. LTED cells are resistant to the combination of antiestrogens and CDK4/6 inhibitors. (A-D) Growth rates of parental cells (MCF7 and T47D) and their derivatives (MCF7-LTED and T47D-LTED) treated with vehicle, 100nM Fulvestrant, 500nM ribociclib or the combination of fulvestrant and ribociclib were measured for 12 days and compared to time=0 (beginning of treatment). Cell number was measured using a Coulter Counter. Points represent the mean ± SE of relative number (normalized to t=0) for a single representative experiment performed in triplicate. * *P*<0.05, ** *P*<0.01, **** *P*<0.0001 (ANOVA). Data presented here is representation of three independent experiments (n=3).

Supplementary Figure S3

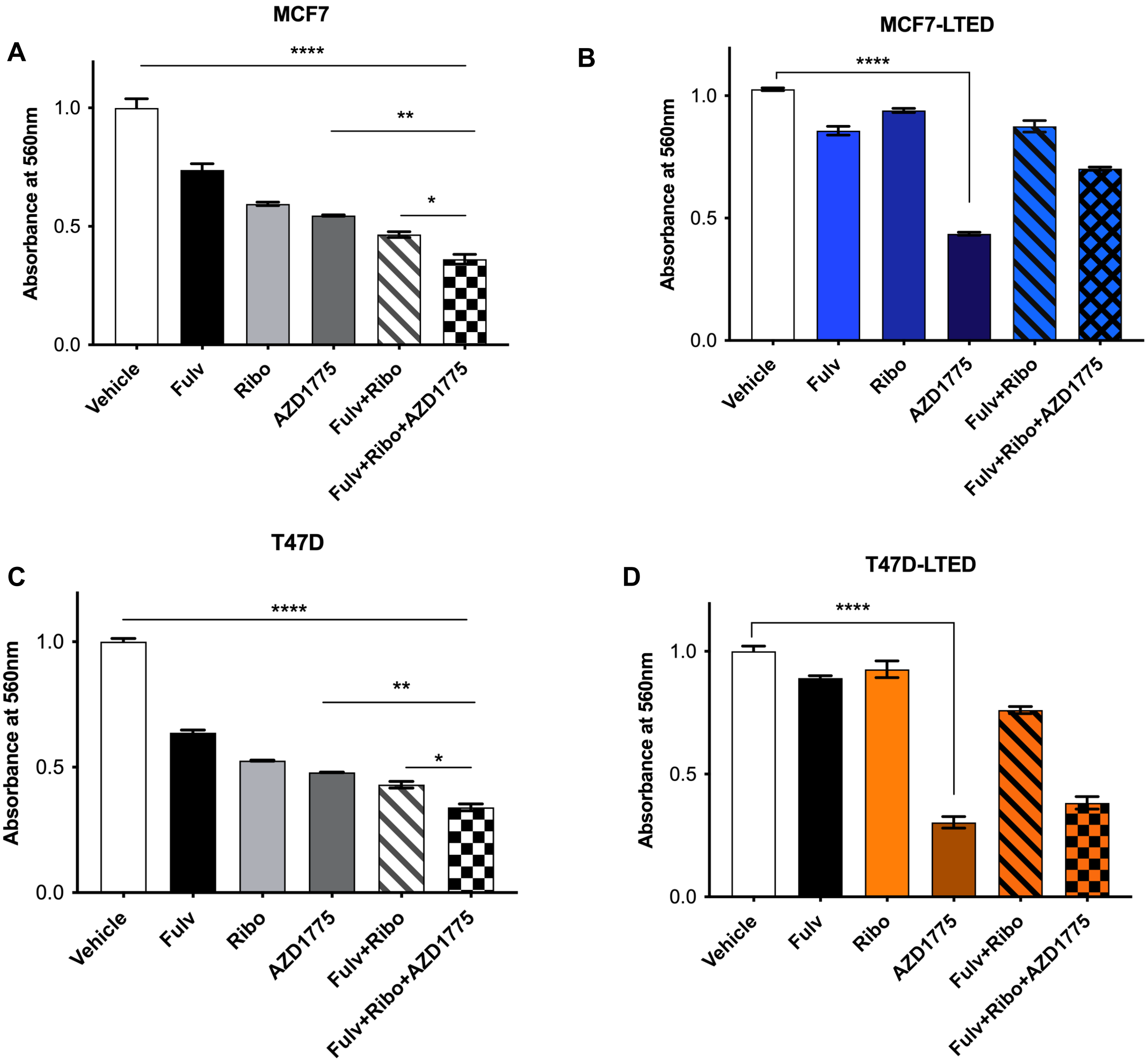

Figure S3. AZD1775 alone suppressed growth of LTED cells. (A-D) Parental MCF7 and T47D cells and their LTED derivatives were treated with vehicle, 100nM fulvestrant (Fulv), 500nM ribociclib (Ribo), 500nM AZD1775, combination of fulvestrant and ribociclib or the combination of fulvestrant, ribociclib and AZD1775 for 6 days. Cell number was measured by crystal violet assays. Bar graph represent the mean ± SE of relative number (normalized to vehicle) for a single representative experiment performed in sextuplicates. **p*<0.05, **p<0.01, *****p*<0.0001 (One-way ANOVA). Data presented here is representation of three independent experiments (n=3).

Supplementary Figure S4

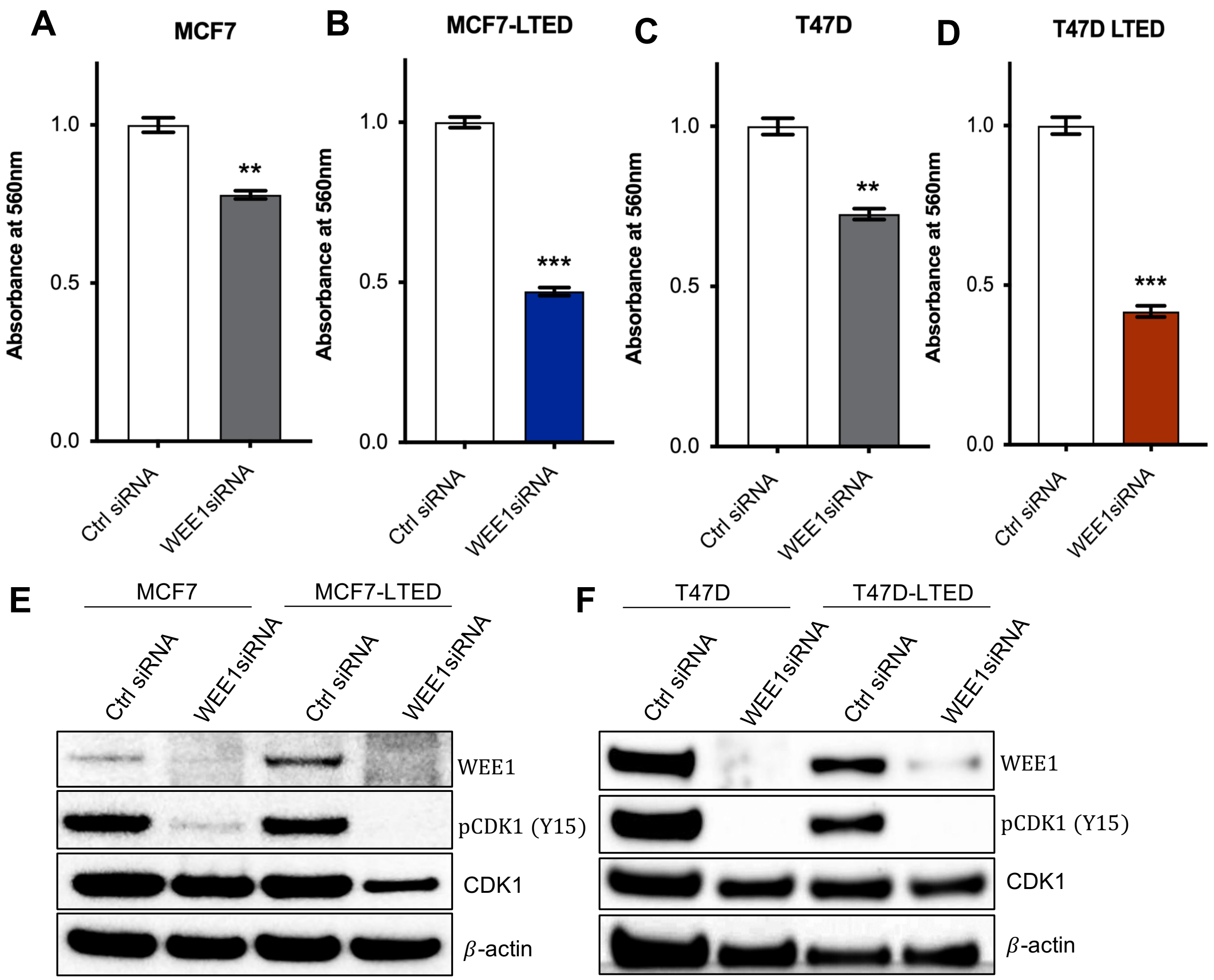

Figure S4. Increased inhibition of cell proliferation in LTED cells following siRNA mediated WEE1 knockdown. MCF7, MCF7-LTED, T47D and T47D-LTED cells were transfected with control (Ctrl) siRNA or WEE1 siRNA for 72 h in 96-well plates for cell number measurements by using crystal violet assays, and in 6-well plates for Western blot analyses. A-D, Bar graph represent the mean ± SE of relative number (normalized to Ctrl) for a representative experiment performed in sextuplicates. **<0.01, ****p*<0.001 (Student's t test). Data presented here is representation of three independent experiments (n=3). E and F, Western blotting of whole cell lysates with WEE1 antibody showed successful downregulation of siRNA mediated knockdown and subsequent decrease in p-CDK1(Y15) in cells transfected with WEE1 siRNA compared with cells transfected with Ctrl siRNA. Note: for T47D-LTED cells, protein loading for Ctrl siRNA and WEE1 siRNA were lower compared with proteins for T47D cells as indicated by β-actin.

Supplementary Figure S5

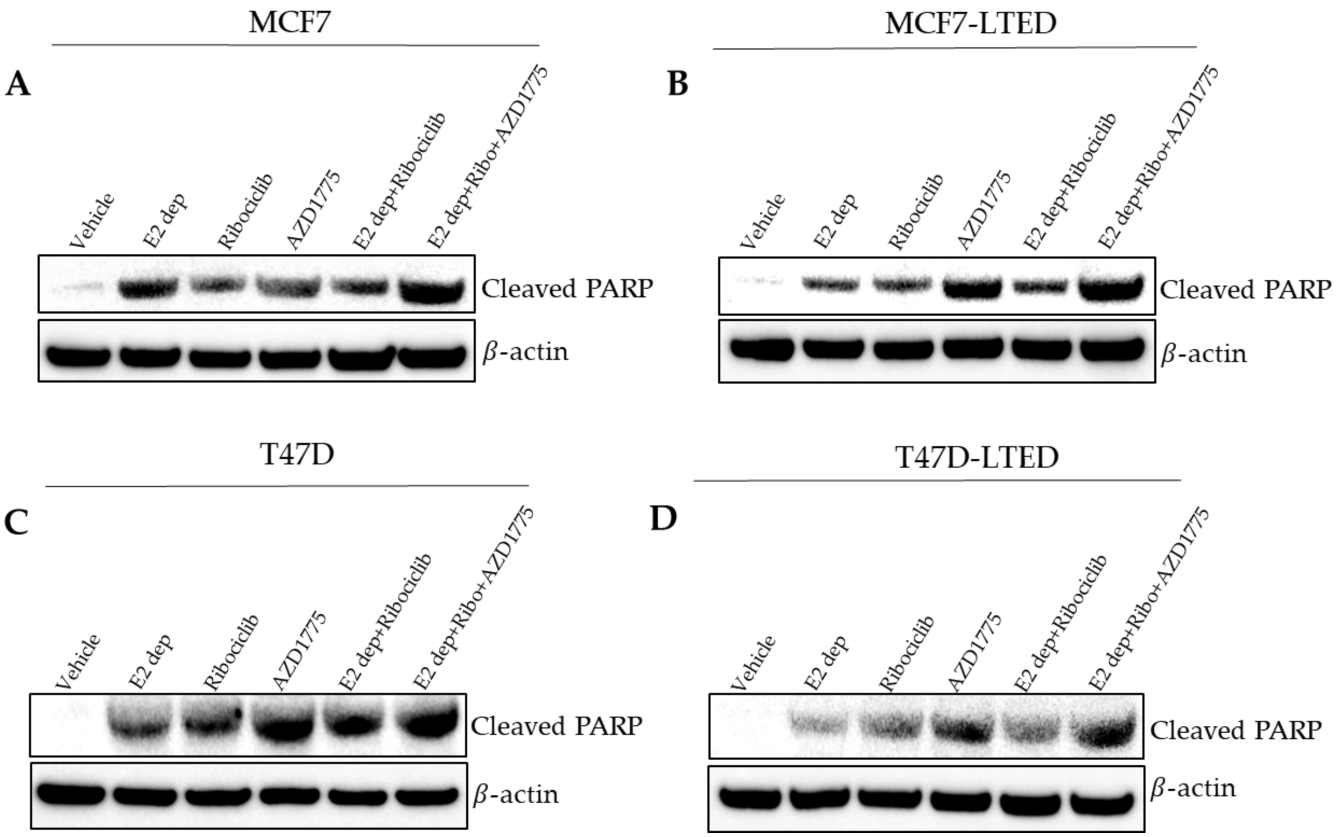

Figure S5. AZD1775 treatment induced PARP cleavage in parental and LTED cells. A-D, Cells were treated with E2 deprivation, 500nM ribociclib, 500nM AZD1775 or the combination of E2 deprivation and ribociclib or the triple combination of E2 deprivation, ribociclib and AZD1775 for 6 days. Whole cell lysates were subjected to immunoblotting with cleaved PARP (Asp214), a marker for apoptosis. Increased levels of cleaved PARP was noted in AZD1775 treated cells in all cells compared to their respective vehicle control treated conditions. Blots are representations from three independent experiments (n=3).
